# Supplementary material for: Viral suppression and associated factors after enhanced adherence counseling among people living with HIV with unsuppressed viral loads at tertiary and first-level health facilities in Zambia: A retrospective cohort study
Source: PLOS Glob Public Health. 2025 Nov 5;5(11):e0005417. doi: 10.1371/journal.pgph.0005417 (PMC12588457; doi:10.1371/journal.pgph.0005417)
Supplement: Attachment — Submitted filename: Response to reviewers .docx [file pgph.0005417.s004.docx]

Rebuttal letter or Response letter to the editor and reviewer

Dear Editor,

PLOS Global Public Health

RE: Response to Reviewers-MANUSCRIPT PGPH-D-25-00977

We would like to thank you and the reviewers for your thoughtful and constructive comments on our manuscript titled "Viral suppression and associated factors after enhanced adherence counselling among people living with HIV unsuppreseed viral loads at tertiary and first level health facilities in Zamiba: a retrospective cohort study", submitted to PLOS Global Public Health.

We have carefully considered all the feedback and have revised the manuscript accordingly. Please find below our detailed responses to each of the reviewers’ comments. We have addressed each point raised and made corresponding changes in the manuscript, which are highlighted in the revised version.

We believe these revisions have significantly improved the quality and clarity of our work. We are grateful for the opportunity to revise and resubmit our manuscript, and we hope that the revised version meets the expectations of the reviewers and the editorial board.

Thank you once again for your time and consideration.

Sincerely,
Chitalu Chanda
University Teaching Hospital
e mail: chtalu@gmail.com
On behalf of all co-authors

Reviewer 1:

Reviewer #1: Thank you for the opportunity to review this manuscript. The manuscript was well written and describes the effectiveness of enhanced adherence counselling among individuals with high HIV viral loads at three months and 12 months post initiation of EAC. The manuscript also describes factors that were associated with achieving viral suppression in the same population of PLHIV. While the paper reads fairly easily there are a number of methodological flaws and inconsistencies in the reporting that the authors need to address. The main one is that it’s not clear what the outcome for the analysis was ie to compare the % virally suppressed post EAC among those who received EAC vs those who did not (implied in the abstract) OR to compare the effectiveness between those who received EAC via telephone only vs those who received EAC in-person and via telephone (as implied and presented in the results). I have listed these and other concerns of these in the comments below

*Response:* Thank you. The outcome of the analysis has been stated. The standard of practice is to ensure that all patients with high viral loads are enrolled in enhanced adherence counseling. The outcome of interest was viral suppression among those enrolled in EAC and who received different modes of EAC (facility, community, and telephone).

Introduction- the authors state the objectives as to describe viral suppression and associated factors without mentioning that this was post EAC. This makes the preceding discussion of EAC seem odd

Response: Thank you for the observation. The correction has been made accordingly.

Results – in the results section the authors reported that EAC delivered both in-person and via the telephone increased the likelihood of viral suppression by 15%. They don’t state at what time point- 3m or 12 m or at either time point and they also don’t state what the comparison group was. Without stating explicitly was the comparison group was it was easy to assume it was vs those who did not receive EAC which didn’t make sense as all people enrolled were rolled at initiation of EAC

Response: Thank you for the comment. The comparative group for the 15% has been stated.. Thank you for the observation, the comparison group for the 6 MMD was indicated (ref group).

The authors concluded that EAC effectively improved and sustained viral suppression again. This seems like overstating the effect. I would say that EAC only modestly improves VL suppression. 15% is not too impressive and there was the question of DTG roll-out in the same period. Also it should say in-person + telephone based improved viral load suppression by 15% compared to telephone alone as this is what the data showed.

Response: The conclusion has been changed to indicate the it was moderately and that the use of telephone modes for conducting EAC improved viral suppression.

Introduction
o In Lines 65 – 68 the authors wrote that EAC was shown to be effective in a achieving viral suppression by 61- 73.8% in the countries listed. Was this under trial conditions?
Response: The conditions of the previous evidence has been corrected. These were observational studies.

Methodology
o In the section on study design and setting, may the authors say a bit more about these hospitals and the services they provided? What are their catchment areas and did they refer to one another? Why pick these two out of all eligible facilities in Lusaka?

*Response*: Thank you for the observation. The facilities were purposively selected as they are two facilities with the largest number of recipients of care in Lusaka.
o In the section on sample size calculations, were there any other assumptions made about the sample size. From the information provided I cannot replicate this sample size calculation. Were the viral suppression levels discussed post EAC? Also what precision level was assumed in the sample size calculations?

*Response:* The sample calculation related to the 95% CI and precision has been included. Thank you.
o Regarding Figure 1, are the numbers in Figure 1 what was found after data collection was completed or what was assumed at study design. If this is what was found, please move to the results. It will be good of the authors can also include a consort diagram in the results

*Response*: Thank you for the advice. The figure has been moved to the results section. Figure 1 was moved to the results section.
o The outcome of interest was VL<200 copies/ml at 3m. This piece of information should be added to the abstract. Also why 200 copies/ ml?

*Response*: Thank you for the comment. This has been included in the abstract. Most studies explored VL at a level of less than 1000. However, most evidence for public health response to control HIV transmission is associated with VL less than 200. Hence, we opted for this level, which has proven to be optimal for HIV control.

• Results
o Line 150 should be areas since there were two facilities

*Response*: The correction has been made to indicate areas

Line 153 - at what point was this CD4 count measured? Also in the setting and background, say something about CD4 count monitoring or measurement in the context of PLHIV on ART and having a high viral load or starting EAC

*Response*: The CD4 time was collected at the time of enrolment into EAC, and this has been corrected.

o Table 1: how does the % of males/ females in this population compare with that of PLHIV in care and on ART?
*Response*: The proportions do compare. In the Treatment current, we have more females than males in care.

o Table 1: regarding mode of EAC, shouldn’t this be categorized as physical only vs physical and telephone. Why add telephone only when there was no- one who received it

*Response*: Table 1 has been corrected as physical only, physical only, and both. We aimed to show the readers that none of the participants received EAC through the telephone only.

o Table 1: regarding baseline CD4 count at enrolment, please see earlier comment. Is this baseline as in the start of ART/ entry into care? What is the policy around CD4 count monitoring and EAC / loss of viral suppression?

*Response*: This is the CD4 at the time of enrolment into EAC. The policy is CD4 every 6 months, thereafter every year. However, those with high viral loads have to have a CD4 count to help guide screening for advanced HIV disease.

o Table 1: what is meant by HIV indexing

Response: This is a public health intervention where HIV testing services are offered to recipients of care with high viral loads. The meaning of the HIV indexing has been included in the footnote of the table.

o Table 1: duration on ART: what is this, median with IQR. If so say so
*Response*: The first row of the table is headed as median with IQR which accounts for this observation.

o Table 4: This table is mixed up. For some variables the authors present column totals and for some they present row totals. Please present column totals for all variables. Alternatively remove table 4 and incorporate the information provided in it in what is table 5 as n/N for each category of variables

*Response*: Thank you for the valuable advice. The table has been revised to present column totals.

o Table 4: I don’t understand the variable place of EAC. Is it for those who got in-person. How was the community based EAC provided and by who? Maybe add to the settings section

Response: The place where EAC was being provided include the facility or the community( at or close to the client’s home. All clients were initially supposed to receive in person however, challenges around COVID 19 era and travel meant some clients had to later get a phone adherence counselling session. This has been included in the setting.

o Line 183: compared to what

*Response*: Currently, 193 has been corrected for the comparison group.

• Discussion
o The so-what of the paper doesn’t come out clearly. As mentioned earlier, telephone + in-person achieved a modest improvement in VL suppression. Given the most effect, was there an understanding of costs involved and if the costs of telephone vs in-person are balanced by the risk of unsuppressed VLs. Was there any data form literature on alternatives to the telephone eg whatsapp chat bots etc

Response: This has been corrected to highlight an opportunity for differentiated models of providing EAC and integrating technology. There no study which enrolled clients using Whatsapp alternatives. This provides for future studies

Reviewer 2:

This manuscript explored factors associated with viral suppression for people living with HIV (PLHIV) post-intervention (Enhanced Adherence Counseling: EAC). Viral suppression was assessed 3- and 12-months post EAC (e.g., mode, place administered). Study findings suggests viral suppression was achieved at 3-months (85%) and sustained at 12-months (96%). EAC delivered through a combination of telephone and in-person sessions increased the likelihood of suppression. It was also noted that enrollment in the 6-month multi-month dispensing (MMD) taking tenofovir/lamivudine/dolutegravir were more likely to achieve suppression. This manuscript is well-written and covers the important topic of viral suppression and targeted intervention to improve and sustain suppression. The study findings can be important when developing interventions to address viral suppression. However, the limited study methodology makes it difficult to determine just how impactful the manuscript will be to the related literature. Given the importance of the intervention (EAC) in relation to the study outcomes, more information needs to be included about EAC as well as other factors believed to impact viral suppression.

Response: Thank you for the comment

1. There is very little information here regarding EAC, specifically: 1) What does the three modes of EAC look like in practice?; 2) What is contained in the EAC sessions?; 3) “Places EAC Given” refers to what exactly?; 4) What is meant by “prior EAC?”; and 5) When referring to time to EAC, are there other interventions during this time or just a waitlist?

*Response*: The methods section has been adjusted to include information on the EAC. The EAC was provided by trained counsellors and given from facility or community. For those not able to attend this session, a telephone option was given.

2. The manuscript briefly mentions 6-month MMD, were other options available?

*Response:* 6 MMD is a differentiated service delivery model given to stable clients. Some of these were clients with suppressed HIV VL less 1000 and on treatment for more than 1 years. Those not getting multi monthly were getting 3 monthly reviews or less and refills. This has been corrected in the manuscript

3. What are the differences between primary and tertiary facilities (specifics can vary for area to area).
   *Response:* The primary is a first-level facility, and the tertiary is a regional referral center. Some clients are referred from the primary facility to the tertiary, and they are in the same location. The catchment area is slightly different, though the facilities have similar numbers for recipients of care.
4. it is noted that the sample is made up of adults age 31 and old (median 39), and given that younger PLHIV (ages 16 - 29) are a population most impacted by the lack of viral suppression, it may be important to specifically note how this study can possibly impact this subgroup.

Response: This has been acknowledged as age especially among young people is associated with lack of viral suppression. Our analysis sexplored the impact of unit change in age increase in relation to achieving viral suppression.

5. Occupation is very important with regard to adherence patterns, and while data was not available, suggestions for future directions is important.

Response: Thank you for the recommendation. The retrospective nature of the data had this as missing variable. The integration of occupation will be considered for the future studies.
