# Supplementary material for: Viral suppression and associated factors after enhanced adherence counseling among people living with HIV with unsuppressed viral loads at tertiary and first-level health facilities in Zambia: A retrospective cohort study
Source: PLOS Glob Public Health. 2025 Nov 5;5(11):e0005417. doi: 10.1371/journal.pgph.0005417 (PMC12588457; doi:10.1371/journal.pgph.0005417)
Supplement: Attachment — Submitted filename: Response to reviewers 15.09.2025 dr chitalu.docx [file pgph.0005417.s005.docx]

Rebuttal letter or Response letter to the editor and reviewer

Dear Editor,

PLOS Global Public Health

RE: Response to Reviewers-MANUSCRIPT PGPH-D-25-00977

We would like to thank you and the reviewers for your thoughtful and constructive comments on our manuscript titled "Viral suppression and associated factors after enhanced adherence counselling among people living with HIV unsuppreseed viral loads at tertiary and first level health facilities in Zamiba: a retrospective cohort study", submitted to PLOS Global Public Health.

We have carefully considered all the feedback and have revised the manuscript accordingly. Please find below our detailed responses to each of the reviewers’ comments. We have addressed each point raised and made corresponding changes in the manuscript, which are highlighted in the revised version.

We believe these revisions have significantly improved the quality and clarity of our work. We are grateful for the opportunity to revise and resubmit our manuscript, and we hope that the revised version meets the expectations of the reviewers and the editorial board.

Thank you once again for your time and consideration.

Sincerely,
Chitalu Chanda
University Teaching Hospital
e mail: chtalu@gmail.com
On behalf of all co-authors

Additional Editor Comments (if provided):

This is a well-written manuscript, and the authors did a good job responding to the questions raised by the reviewers. However, there are still issues to be resolved:

1. To enhance the quality of reporting of a cohort study, it is recommended to use the STROBE for the cohort study checklist (<https://www.strobe-statement.org/checklists/>). Please fill out the form and add it to the manuscript as supplementary materials. And please address the relevant items, if possible, in the text.

Response: thank you for the advice , we have attached the strobe checklist

2. A comment: The adjusted analysis has the issue of using p-values to choose what to adjust for. This is a bad practice when the goal is to find potential factors. And if it is done, the p-value (to choose variables to adjust for) should not be so stringent. Nevertheless, it appears that age and sex were forced into the model (which is good!).
A question: And why is “age” included as a linear covariate? This means its “effect” is expected to be the same for the range of ages. Is that plausible?

Response: We thank the reviewer for this important observation. We what to see a unit increase in age and how it affects the outcome that the reason why we left as linear covariate. To select variables we also selected variables into the final model based previous literature as illustrated in the data analysis plan of the manuscript section

3. We need a good paragraph, either in the introduction or in the methods, explaining what EAC consists of in Zambia. One reviewer asked for this already.

Response: The methods section has been adjusted to include information on the EAC. The EAC was provided by trained counsellors and given from facility or community. For those not able to attend this session, a telephone option was given as shown in line 98- 105.

4. Please Cite REDCap and Stata.

Response: thank you. we have cited REDcap and Sata

5. Table 1 - Should be only for baseline, i.e, characteristics at enrolment to EAC. Please revise.
- What is “HIV indexing done”?

Response: The HIV index testing is done by having HIV test done for partners and close family members of the positive clients. The meaning of the HIV indexing has been included in the footnote of the table.

6. Table 2 - better be presented as a figure with three boxplots (and the Y-axis in logarithmic scale, add 0.5 before the logarithm). Move table 2 to the annexes.

Response: thanks, very much for the suggestion. We have now represented table 2 into a box plot. And table has been added as a supplementary

7. Table 3 - Add a note explaining how the proportions are calculated

## Response: thank you for the suggestion . Table 3 has now become table 2 . We have added a foot note explaining how they were calculated and additionally, the calculation of proportions is explained below the subheading “ HIV Viral Suppression After EAC”.

8. Table 4:
- Add a column with prevalence of “Viral suppression after EAC”
- We do not need p-values in this table, especially with almost the same p-values on the unadjusted analysis in table 5.
- Add a footnote explaining how the proportions are computed

Response: thank you . table 4 has been completely removed , it is unadjusted analysis which is in table 3 which is former table 5.

**Comments to the Author**

1. If the authors have adequately addressed your comments raised in a previous round of review and you feel that this manuscript is now acceptable for publication, you may indicate that here to bypass the “Comments to the Author” section, enter your conflict of interest statement in the “Confidential to Editor” section, and submit your "Accept" recommendation.

Reviewer #1: All comments have been addressed

2. Does this manuscript meet PLOS Global Public Health’s [publication criteria](https://track.editorialmanager.com/CL0/https:%2F%2Fjournals.plos.org%2Fglobalpublichealth%2Fs%2Fcriteria-for-publication/1/010f0198ebc8bc08-f11f5e5f-1b94-4a7b-a616-05681a1fbf0b-000000/JFfJQuaakhdWKRJhTVSXwF1ehOjK4rvYtCp-dLO8Vvs=226)? Is the manuscript technically sound, and do the data support the conclusions? The manuscript must describe methodologically and ethically rigorous research with conclusions that are appropriately drawn based on the data presented.

Reviewer #1: Yes

3. Has the statistical analysis been performed appropriately and rigorously?

Reviewer #1: Yes

4. Have the authors made all data underlying the findings in their manuscript fully available (please refer to the Data Availability Statement at the start of the manuscript PDF file)?

The [PLOS Data policy](https://track.editorialmanager.com/CL0/https:%2F%2Fjournals.plos.org%2Fglobalpublichealth%2Fs%2Fdata-availability/1/010f0198ebc8bc08-f11f5e5f-1b94-4a7b-a616-05681a1fbf0b-000000/N3EMn_B0BIgsU0o3J7EOlFcHMEjYOlju6aSQ1fglXzA=226) requires authors to make all data underlying the findings described in their manuscript fully available without restriction, with rare exception. The data should be provided as part of the manuscript or its supporting information, or deposited to a public repository. For example, in addition to summary statistics, the data points behind means, medians and variance measures should be available. If there are restrictions on publicly sharing data—e.g. participant privacy or use of data from a third party—those must be specified.

Reviewer #1: No

5. Is the manuscript presented in an intelligible fashion and written in standard English?

PLOS Global Public Health does not copyedit accepted manuscripts, so the language in submitted articles must be clear, correct, and unambiguous. Any typographical or grammatical errors should be corrected at revision, so please note any specific errors here.

Reviewer #1: Yes

6. Review Comments to the Author

Please use the space provided to explain your answers to the questions above. You may also include additional comments for the author, including concerns about dual publication, research ethics, or publication ethics. (Please upload your review as an attachment if it exceeds 20,000 characters)

Reviewer #1: Thank you for the opportunity to re-review this manuscript. The authors did a good job of addressing the comments I had. I am still not clear how the sample size was determined. I assumed viral suppression of 61% +/- 5% at 3 months, an alpha level of 5% and 80% power. This gives a sample size of 189 using the Score test method. If the sample size is inflated by 10%, it still doesnt give 386. Maybe the authors can calculate power for the realized sample size instead

7. PLOS authors have the option to publish the peer review history of their article ([what does this mean?](https://track.editorialmanager.com/CL0/https:%2F%2Fjournals.plos.org%2Fglobalpublichealth%2Fs%2Feditorial-and-peer-review-process%23loc-peer-review-history/1/010f0198ebc8bc08-f11f5e5f-1b94-4a7b-a616-05681a1fbf0b-000000/RkffAESAJj5hgNPFhsAygmSlVBUoRMBsufTADrxwSgY=226)). If published, this will include your full peer review and any attached files.


**Do you want your identity to be public for this peer review?** If you choose “no”, your identity will remain anonymous but your review may still be made public.


For information about this choice, including consent withdrawal, please see our [Privacy Policy](https://track.editorialmanager.com/CL0/https:%2F%2Fwww.plos.org%2Fprivacy-policy/1/010f0198ebc8bc08-f11f5e5f-1b94-4a7b-a616-05681a1fbf0b-000000/zqDl0RVW_LvGbovJ-Gl3CHOA6L9SXhHOu9ottEq9QJU=226).

Reviewer #1: No
